# Frontotemporal-spectrum disorders and functional independence in non-demented ALS patients

**DOI:** 10.1007/s10072-023-07074-3

**Published:** 2023-09-29

**Authors:** Edoardo Nicolò Aiello, Federica Solca, Silvia Torre, Francesco Gentile, Francesco Scheveger, Marco Olivero, Eleonora Colombo, Alessio Maranzano, Martina Manzoni, Claudia Morelli, Alberto Doretti, Federico Verde, Vincenzo Silani, Nicola Ticozzi, Barbara Poletti

**Affiliations:** 1 Department of Neurology and Laboratory of Neuroscience, IRCCS Istituto Auxologico Italiano, Piazzale Brescia 20, 20149 Milan, MI Italy; 2 Neurology Residency Program, Università Degli Studi Di Milano, Milan, Italy; 3 Child Psychopathology Unit, Scientific Institute, IRCCS E. Medea – La Nostra Famiglia, Bosisio Parini, Lecco, Italy; 4 Department of Pathophysiology and Transplantation, ”Dino Ferrari” Center, Università degli Studi di Milano, Milan, Italy; 5 Department of Oncology and Hemato-Oncology, Università degli Studi di Milano, Milan, Italy

**Keywords:** Amyotrophic lateral sclerosis, Activities of daily living, Neuropsychology, Functional independence, Frontotemporal degeneration

## Abstract

**Background:**

The present study aimed at determining whether, net of motor confounders, neuropsychological features affect functional independence (FI) in activities of daily living (ADLs) in non-demented amyotrophic lateral sclerosis (ALS) patients.

**Methods:**

*N* = 88 ALS patients without frontotemporal dementia were assessed for FI—Katz’s Basic ADL Scale (BADL) and Lawton-Brody’s Instrumental ADL Scale (IADL)—, cognition—Edinburgh Cognitive and Behavioural ALS Screen (ECAS)—and behaviour—Beaumont Behavioural Inventory and Dimensional Apathy Scale. The association between cognitive and behavioural measures and BADL/IADL scores was assessed by covarying for demographics, anxiety and depression levels, disease duration and motor confounders—i.e. ALS Functional Rating Scale-Revised (ALSFRS-R) scores, progression rate and both King’s and Milano-Torino stages.

**Results:**

Higher scores on the ECAS-Language were associated with higher IADL scores (*p* = 0.005), whilst higher apathetic features—as measured by the Dimensional Apathy Scale (DAS)—were inversely related to the BADL (*p* = 0.003). Whilst IADL scores were related to all ECAS-Language tasks, the DAS-Initiation was the only subscale associated with BADL scores. Patients with abnormal ECAS-Language (*p* = 0.023) and DAS (*p* = 0.008) scores were more functionally dependent than those without.

**Discussion:**

Among non-motor features, language changes and apathetic features detrimentally affect FI in non-demented ALS patients.

## Background

Frontotemporal-spectrum disorders (FTSDs) are acknowledged to detrimentally affect survival in non-demented amyotrophic lateral sclerosis (ALS) patients [1] by interfering with decision-making and adherence within care settings [2, 3].

However, little is known on the extent to which neuropsychological features impact on patients’ functional independence (FI) in daily living—likely due to their physical disabilities representing a major confounder to the study of such a matter [4, 5]. Only two reports have indeed to this day addressed this topic—the first, by Mioshi et al. [4], showing that FI was dependent on both motor and behavioural features, and the second, by Kapustin et al. [5], failing to detect an association between cognitive/behavioural features and FI net of ALS severity. However, these studies either preceded the availability of [4], or did not employ [5], ALS-specific cognitive/behavioural measures [6]. Moreover, the only study [5] having explored the association between FI and a performance-based measure of cognition did not provide single domain-level information.

The above being said, assessing how neuropsychological features impact FI in both basic and instrumental activities of daily living (ADL) in this population is prognostically pivotal, as it would shed further light on the ecological relevance of FTSDs in ALS besides their already acknowledged impact on survival [1, 2]. Hence, by employing a detailed and comprehensive set of ALS-specific cognitive and behavioural measures, the present study aimed at determining whether, net of motor confounders, FTSDs affect FI in non-demented ALS patients.

## Methods

### Participants

Eighty-eight ALS patients [7] consecutively referred to IRCCS Istituto Auxologico Italiano, Milano, Italy, between 2020 and 2023 were recruited. Patients did not present with (1) a co-morbid diagnosis of frontotemporal dementia (FTD) [8, 9], (2) ALS-unrelated neurological/psychiatric disorders, (3) severe/unstable general-medical conditions and (4) uncorrected sensory deficits.

### Materials

FI was assessed via the Basic Activities of Daily Living Scale (BADL) by Katz et al. [10]—ranging 0–6 and assessing basic ADL—and the Instrumental Activities of Daily Living Scale (IADL) by Lawton and Brody [11]—ranging 0–8 and assessing instrumental ADL. Whenever at least one item on the IADL was not applicable, a proportion out of the applicable maximum was computed so that patients’ scores could be comparable among each other. Such “adjusted” IADL scores were computed by multiplying by 8 individual IADL scores weighted on their applicable maximum. The result of this computation was then rounded up or down the nearest integer if the first decimal digit was ≥ 0.50. For example, given an applicable maximum of 7 (i.e. one item not being applicable) and an actual score of 5, the “adjusted” IADL score—computed as (5/7)*8—is equal to 5.71—and thus 6 when rounded up. This adjustment was performed for 45 patients.

Cognition was assessed via the cognitive section of the Edinburgh Cognitive and Behavioural ALS Screen (ECAS) [12]—comprising 5 performance-based subscales tapping on Language (ECAS-L; range = 0–28), Fluency (ECAS-F; range = 0–24), Executive functioning (ECAS-EF; range = 0–48), Memory (ECAS-M; range = 0–24) and Visuospatial abilities (ECAS-VS; range = 0–12)—whilst behaviour with the Beaumont Behavioural Inventory (BBI) [13]—a 41-item, caregiver-report questionnaire covering the full spectrum of ALS patients’ behavioural phenotype (range = 0–123)—and the Dimensional Apathy Scale (DAS) [14]—a 24-item, self-report questionnaire assessing both cognitive and behavioural apathetic features (range = 0–72) and comprising three subscale tapping on dysexecutive features (DAS-Executive), affective disintegration (DAS-Emotional) and reduced cognitive/behavioural initiation (DAS-Initiation). Additionally, anxiety and depression levels were assessed via the State- and Trait-Anxiety Inventory-Form Y (STAI-Y1/-Y2) [15] and the Beck Depression Inventory (BDI) [16], respectively.

Motor status was assessed via the ALS Functional Rating Scale-Revised (ALSFRS-R) [17], progression rate (ΔFS) was computed according to Kimura et al. [18]—i.e. as (48-ALSFRS-R)/disease duration in months—and disease stage was retrieved based on both King’s [19] and Milano-Torino (MiToS) [20] systems.

### Statistics

Both BADL and IADL scores were featured by a moderate ceiling effect and thus did not distribute normally—as indexed by an excessive, negative skewness value (i.e. >|1|) [21], a significant Shapiro–Wilk’s statistics (*p* < 0.001) and visual abnormalities within its histogram and Q-Q plot. Hence, non-parametric techniques and generalized linear models were employed for testing associations and predictions of interest, respectively.

First, the association between the BADL/IADL scores and both cognitive (i.e. ECAS-L, -F, -EF, -M and -VS scores) and behavioural measures (i.e. BBI and DAS scores) was preliminarily explored via Bonferroni-corrected Spearman’s coefficients that partialled out demographics—i.e. sex, age and education—, disease duration (in months), motor status (i.e. ALSFRS-R and ΔFS scores, King’s and MiToS stages) and psychopathological features (i.e. STAI-Y1/-Y2 and BDI scores).

Then, those cognitive and behavioural variables that proved to be significantly related to BADL/IADL scores within these correlational analyses were entered, along with the abovementioned covariates, into a negative binomial regression (NBR) which addressed, as the outcome, a reversed score on the BADL and IADL (rBADL; rIADL)—computed by subtracting from the theoretical maximum (i.e. 6 and 8, respectively) the actual score on the scale at hand. Such an expedient has been employed in order for BADL/IADL scores to adhere to the underlying count-like, and thus right-skewed, distribution which is modelled by the NBR [22]—by, at the same time, not undermining its original metric (since rBADL/rIADL scores reflect the degree of dependence). Within these NBRs, collinearity was diagnosed in the presence of a variance inflation factor (VIF) > 10 and of a tolerance index (TI) < 0.10.

Analyses were run via IBM® SPSS® Statistics (IBM Corp., 2021) and jamovi 2.3 (the jamovi project, 2022). Within the correlational set, missing data points were excluded pairwise, whilst, within the NBRs, a listwise deletion procedure was applied.

## Results

Table 1 summarizes patients’ background and clinical measures.

**Table 1:** Patients’ demographic, clinical and cognitive measures

*N*	88
Sex (male/female)	54/34
Age (years)	64.1 ± 10.8 (30–84)
Education (years)	11.9 ± 4.3 (5–19)
Disease duration (months)	16.6 ± 18. (2–108)
ALSFRS-R	39.4 ± 5.9 (21–48)
ΔFS	0.8 ± 0.8. (0–5.2)
NIV (%)	1%
PEG (%)	0%
Genetics (*N*)	
*C9orf72*/*TARDBP*/*SOD1*	3/1/1
King’s (%)	
Stage 1/2/3/4	39/33/27/1%
MiToS (%)	
Stage 0/1/2	73.3/23/5%
ECAS	
Total	98.5 ± 20.4 (39–127)
Impaired (%)	33%
Language	23.4 ± 3.8 (14–28)
Impaired (%)^a^	23.9%
Fluency	16.5 ± 5.7 (0–24)
Impaired (%)^a^	19.3%
Executive functioning	32.8 ± 9.0 (7–46)
Impaired (%)^a^	25%
Memory	14.4 ± 5.1 (1–21)
Impaired (%)^a^	26.1%
Visuospatial	11.1 ± 1.5 (5–12)
Impaired (%)^a^	13.6%
STAI-Y1	54.7 ± 11 (34–81)
STAI-Y2	48.6 ± 9.4 (33–73)
BDI	13.3 ± 9.1 (0–37)
BBI	2.7 ± 3.1. (0–13)
Abnormal (%)^b^	2%
DAS	22.3 ± 7.6. (5–40)
Abnormal (%)^c^	27%
BADL	5.2 ± 1.4 (1–6)
IADL*	6.4 ± 2.1 (0–8)

*ALS*, amyotrophic laterals sclerosis; *ALSFRS-R*, ALS Functional Rating Scale-Revised; *BADL*, Basic Activities of Daily Living; *BBI*, Beaumont Behavioural Inventory; *BDI*, Beck Depression Inventory; *DAS*, Dimensional Apathy Scale; *ΔFS*, progression rate; *ECAS*, Edinburgh Cognitive and Behavioural ALS Screen; *IADL*, Instrumental Activities of Daily Living; *FII*, Functional Independence Index; *MiToS*, Milano-Torino Staging; *NIV*, non-invasive ventilation; *PEG*, percutaneous endoscopic gastrostomy; *STAI-Y1*, State- and Trait-Anxiety Inventory-Form Y-State-Anxiety; *STAI-Y2*, State- and Trait-Anxiety Inventory-Form Y-Trait-Anxiety. ^a^Poletti et al. [12]; ^b^Iazzolino et al. [13]; ^c^Santangelo et al. [14]; *whenever at least one item on this scale was not applicable, a proportion of out of the applicable maximum was computed for comparability reasons

The results of correlational analyses are reported in Table 2. Net of demographics, disease duration, motor status and psychopathological features, the only associations surviving Bonferroni’s correction (i.e. *α*_*adjusted*_ = 0.007) were those between (1) IADL scores and the ECAS-L (positive coefficient) and (2) BADL scores and the DAS (negative coefficient).

**Table 2:** Spearman’s partial correlation coefficients between BADL/IADL scores and cognitive/behavioural measures

Measure		BADL	IADL
ECAS-Language	*r*_*s*_	0.19	0.32*
	*p*	0.103	0.005
ECAS-Fluency	*r*_*s*_	0.11	− 0.03
	*p*	0.370	0.827
ECAS-Executive	*r*_*s*_	0.19	0.25
	*p*	0.099	0.035
ECAS-Memory	*r*_*s*_	− 0.13	0.02
	*p*	0.253	0.849
ECAS-Visuospatial	*r*_*s*_	0.02	0.18
	*p*	0.880	0.136
BBI	*r*_*s*_	− 0.16	− 0.22
	*p*	0.186	0.067
DAS	*r*_*s*_	− 0.35*	− 0.10
	*p*	0.003	0.417

*BADL*, Basic Activities of Daily Living; *BBI*, Beaumont Behavioural Inventory; *DAS*, Dimensional Apathy Scale; *ECAS*, Edinburgh Cognitive and Behavioural ALS Screen; *IADL*, Instrumental Activities of Daily Living. These analyses were run by partialling out the following variables: age, education, sex, disease duration (in months), ALS Functional Rating Scale-Revised scores, progression rate, Milano-Torino and King’s scores, State- and Trait-Anxiety Inventory-Form Y1/Y2 and Beck Depression Inventory scores. *Significant coefficient at *α*_*adjusted*_ = 0.007

Accordingly, two NBRs were run: the first, addressing rIADL scores as the outcome and the ECAS-L as the predictor; the second, addressing rBADL scores as the outcome and the DAS as the predictor. The results of these models are shown in Table 3. No collinear regressors were detected within either the model addressing the ECAS-L (VIF ≤ 5.43; TI ≥ 0.18) or that addressing the DAS (VIF ≤ 5.55; TI ≥ 0.18). Both models were in agreement with the previous correlational analyses—with the ECAS-L and the DAS being predictive of rIADL and rBADL scores, respectively. As to covariates, within both models, lower age and lower ALSFRS-R scores were also associated a higher degree of functional dependence; additionally, within the model addressing the rBADL, female sex, higher MiToS scores and lower STAI-Y2 scores were associated with a poorer FI.

**Table 3:** Results of the NBRs addressing rIADL scores as the outcome and the ECAS-L as the predictor (upper panel) and rBADL scores as the outcome and the DAS as the predictor (lower panel)

Outcome	Independent variable	Slope	*b*	*SE*	OR	95% CI for the OR		*z*	*p*
						LL	UL		
rIADL	Age (years)	–	− 0.03	0.01	0.97	0.94	0.99	− 2.36	0.018
	Education (years)	–	− 0.01	0.04	0.99	0.91	1.07	− 0.34	0.735
	Sex	F vs. M	0.02	0.30	1.02	0.56	1.84	0.05	0.959
	Disease duration (months)	–	− 0.01	0.01	0.99	0.96	1.01	− 1.09	0.274
	ALSFRS-R	–	− 0.10	0.05	0.90	0.81	1.00	− 2.06	0.040
	ΔFS	–	− 0.09	0.24	0.91	0.58	1.47	− 0.39	0.693
	King’s scores	–	0.12	0.20	1.13	0.75	1.69	0.60	0.548
	MiToS scores	–	0.67	0.43	1.94	0.87	4.43	1.56	0.118
	STAI-Y1	–	− 0.02	0.02	0.98	0.95	1.01	− 1.56	0.119
	STAI-Y2	–	0.02	0.02	1.02	0.98	1.05	1.00	0.315
	BDI	–	− 0.01	0.02	0.99	0.95	1.04	− 0.33	0.743
	ECAS-Language	–	− 0.09	0.04	0.91	0.84	0.99	− 2.37	0.018
rBADL	Age (years)	–	− 0.07	0.02	0.93	0.90	0.97	− 3.64	< 0.001
	Education (years)	–	− 0.08	0.04	0.93	0.85	1.01	− 1.80	0.073
	Sex	F vs. M	0.83	0.38	2.30	1.09	4.92	2.18	0.029
	Disease duration (months)	–	− 0.03	0.02	0.97	0.92	1.01	− 1.21	0.225
	ALSFRS-R	–	− 0.15	0.05	0.86	0.78	0.95	− 3.10	0.002
	ΔFS	–	− 0.28	0.21	0.75	0.48	1.12	− 1.33	0.183
	King’s scores	–	− 0.03	0.23	0.98	0.62	1.52	− 0.11	0.913
	MiToS scores	–	1.78	0.45	5.93	2.47	14.77	3.94	< 0.001
	STAI-Y1	–	− 0.01	0.02	0.99	0.96	1.03	− 0.34	0.734
	STAI-Y2	–	− 0.09	0.03	0.92	0.86	0.97	− 2.96	0.003
	BDI	–	− 0.01	0.02	0.99	0.95	1.03	− 0.37	0.713
	DAS	–	0.11	0.03	1.12	1.05	1.19	3.69	< 0.001

*ALSFRS-R*, ALS Functional Rating Scale-Revised; *rBADL*, reversed Basic Activities of Daily Living score; *BBI*, Beaumont Behavioural Inventory; *BDI*, Beck Depression Inventory; *DAS*, Dimensional Apathy Scale; *ΔFS*, progression rate; *ECAS*, Edinburgh Cognitive and Behavioural ALS Screen; *rIADL*, reversed Instrumental Activities of Daily Living score; *MiToS*, Milano-Torino Staging; *STAI-Y1*, State- and Trait-Anxiety Inventory-Form Y-State-Anxiety; *STAI-Y2*, State- and Trait-Anxiety Inventory-Form Y-Trait-Anxiety. Significant *p* values are in bold

Notably, when re-running the same NBRs by substituting the ECAS-L and the DAS with their respective below- vs. above-cutoff scores [12, 14], such predictors retained their significance (Table 4)—with patients performing defectively on the ECAS-L (23.9%) being more functionally dependent on the rIADL (*M* = 2.24; *SE* = 0.60) than those performing within the normal range (*M* = 1.08; *SE* = 0.24), and patients with an above-cutoff DAS score (27%) being more functionally dependent on the rBADL (*M* = 1.12; *SE* = 0.43) than those with a below-cutoff score on this scale (*M* = 0.38; *SE* = 0.11).

**Table 4:** Results of the NBRs addressing rIADL scores as the outcome and impaired vs. unimpaired ECAS-L scores as the predictor (upper panel) and rBADL scores as the outcome and above- vs. below-cutoff DAS scores as the predictor (lower panel)

Outcome	Independent variable	Slope	*b*	*SE*	OR	95% CI for the OR		*z*	*p*
						LL	UL		
rIADL	Age (years)	–	− 0.03	0.01	0.97	0.94	1.00	− 2.27	0.023
	Education (years)	–	− 0.04	0.04	0.96	0.89	1.04	− 1.05	0.294
	Sex	F vs. M	− 0.00	0.30	1.00	0.55	1.81	− 0.00	0.997
	Disease duration (months)	–	− 0.02	0.01	0.98	0.96	1.01	− 1.29	0.198
	ALSFRS-R	–	− 0.10	0.05	0.90	0.81	1.00	− 2.06	0.039
	ΔFS	–	− 0.10	0.23	0.90	0.58	1.47	− 0.43	0.666
	King’s scores	–	0.10	0.20	1.11	0.74	1.67	0.51	0.610
	MiToS scores	–	0.68	0.42	1.98	0.89	4.50	1.62	0.104
	STAI-Y1	–	− 0.03	0.02	0.97	0.94	1.00	− 1.75	0.081
	STAI-Y2	–	0.02	0.02	1.02	0.99	1.06	1.26	0.207
	BDI	–	− 0.00	0.02	1.00	0.96	1.04	− 0.10	0.917
	ECAS-Language	Unimpaired vs. impaired	− 0.73	0.32	0.48	0.25	0.93	− 2.28	0.023
rBADL	Age (years)	–	− 0.07	0.02	0.93	0.90	0.97	− 3.65	< 0.001
	Education (years)	–	− 0.08	0.04	0.92	0.85	1.00	− 1.91	0.056
	Sex	F vs. M	0.36	0.34	1.43	0.73	2.82	1.05	0.293
	Disease duration (months)	–	− 0.03	0.02	0.97	0.92	1.01	− 1.20	0.230
	ALSFRS-R	–	− 0.12	0.04	0.88	0.81	0.97	− 2.74	0.006
	ΔFS	–	− 0.12	0.20	0.89	0.59	1.29	− 0.61	0.542
	King’s scores	–	0.02	0.22	1.02	0.66	1.57	0.09	0.931
	MiToS scores	–	1.84	0.46	6.32	2.58	16.10	3.98	< 0.001
	STAI-Y1	–	0.00	0.02	1.00	0.97	1.03	0.04	0.972
	STAI-Y2	–	− 0.04	0.02	0.96	0.92	1.00	− 1.87	0.061
	BDI	–	− 0.02	0.02	0.98	0.94	1.02	− 1.04	0.300
	DAS	Below- vs. above-cutoff	− 1.15	0.43	0.32	0.13	0.74	− 2.64	0.008

*ALSFRS-R*, ALS Functional Rating Scale-Revised; *rBADL*, reversed Basic Activities of Daily Living score; *BBI*, Beaumont Behavioural Inventory; *BDI*, Beck Depression Inventory; *DAS*, Dimensional Apathy Scale; *ΔFS*, progression rate; *ECAS*, Edinburgh Cognitive and Behavioural ALS Screen; *rIADL*, reversed Instrumental Activities of Daily Living score; *MiToS*, Milano-Torino Staging; *STAI-Y1*, State- and Trait-Anxiety Inventory-Form Y-State-Anxiety; *STAI-Y2*, State- and Trait-Anxiety Inventory-Form Y-Trait-Anxiety. Significant *p* values are in bold

In order to exploratively appraise which task(s) of the ECAS-L were associated with the IADL, a Spearman’s correlational set was addressed, which covaried for demographics (i.e. age, education and sex), disease duration and motor status (i.e. ALSFRS-R, ΔFS, King’s and MiToS scores). Such Spearman’s coefficients revealed that IADL score were related to all ECAS-L tasks—i.e. Naming (*r*_*s*_(88) = 0.25; *p* = 0.025), Comprehension (*r*_*s*_(88) = 0.26; *p* = 0.021) and Spelling (*r*_*s*_(88) = 0.23; *p* = 0.041).

Consistently, the same explorative set of Spearman’s coefficient were run between BADL scores and DAS subscales—by nevertheless also adding STAI-Y1/-Y2 and BDI scores as covariates, given the relevance of such psychiatric features to apathy [23]. These analyses revealed that the BADL was selectively associated with the DAS-Initiation (*r*_*s*_(80) =  − 0.28; *p* = 0.019), whilst not with DAS-Executive (*r*_*s*_(80) =  − 0.17; *p* = 0.159) and -Emotional scores (*r*_*s*_(80) =  − 0.11; *p* = 0.354).

## Discussion

The present study provides relevant insights into the role of FTSDs towards FI in non-demented ALS patients, suggesting that language deficits and apathetic features detrimentally affect everyday-life functioning in this population. More specifically, language dysfunctions—as assessed by the dedicated ECAS subscale—herewith proved to be linked to worse IADL scores, whilst a diminished cognitive-behavioural initiation—as assessed by the Initiation subscale of the DAS—was related to worse BADL scores.

To the best of the authors’ knowledge, this is the first study addressing such a topic by (1) simultaneously encompassing a wide range of both motor and non-motor variables and (2) employing ALS-specific measures of both cognition and behaviour—this granting a sufficiently high level of generalizability to the findings herewith reported.

Overall, the current study aligns with Mioshi et al.’s [4] findings as to the fact that not only motor, but also extra-motor features affect FI in ALS—thus not supporting Kapustin et al.’s [5] recent investigation, where ALSFRS-R scores happened to be the only predictor of FI measures in this population.

The present finding of apathetic features being linked to a lower degree of FI in ALS is consistent with Mioshi et al.’s [4] results—where the Motivation subscale of the Cambridge Behavioural Inventory-Revised [24] proved to be, along with its Abnormal behaviour subscale and ALSFRS-R-Spinal scores, a significant predictor of the Disability Assessment of Dementia [25]. In addition, the current report sheds a further light on the link between apathy and FI in ALS, suggesting that a specific component of this syndrome—i.e. a reduced cognitive-behavioural initiation—impacts on basic ADL in this population. Whilst such a finding is unprecedented within the literature concerning apathy in ALS [25], a cumulative effect of initiation deficits to motor disabilities might be postulated in order to account for it: otherwise said, in patients with a greater decrease in goal-directed activity, motor disabilities might have impacted even more on their FI in basic ADL. Whilst such a hypothesis is of course speculative and thus needs to be further tested, this report still happens align with and add up to the current knowledge on the adverse effect that apathy exerts on ALS patients’ prognosis [25]. Moreover, it is worth mentioning that the current results are also consistent with the literature revealing a detrimental effect of apathetic features towards FI in order neurodegenerative disorders—such as Alzheimer’s, Parkinson’s and Huntington’s disease, as well as frontotemporal degeneration [27–30].

Relevantly, and unprecedentedly when compared to previous studies on the topic [4, 5], this report suggest that FI in instrumental ADLs also depends on cognition, and specifically on language, in ALS. Interestingly, such a finding happens to be unparalleled by the relevant literature addressing other neurodegenerative conditions—such as Alzheimer’s, Parkinson’s and Huntington’s disease, as well as frontotemporal degeneration [31–38]—, where behavioural disturbances, as well as deficits within the executive domain, are rather associated with impaired FI. Nevertheless, this result is per se not surprising—since language impairment (LI) might have easily undermined patients’ communicative and comprehension skills, thus in turn reducing their FI in cognitive-driven ADL (such as those tapped onto by the IADL). After all, the impact of LI on FI in primary language disorders—such as post-stroke aphasia [39] or primary progressive aphasia (PPA) [40]—is widely acknowledged: hence, it is reasonably expected that also mild-to-moderate language deficits, albeit not resulting in a full-blown aphasic syndrome, detrimentally affect FI in instrumental ADL in this population too.

The present study also sheds a light, for the first time, on the ecological relevance of LI in this population. LI within the spectrum of PPA [41–44] occurs in ≈23% of non-demented ALS patients [45], with its detection being also sufficient to classify patients as cognitively impaired according to Strong et al.’s [1] revised criteria for FTSDs in ALS. Moreover, the incidence of LI in this population has been reported to increase over time [46]. Adding up to such stances, findings herewith reported highlight that LI in non-demented ALS patients is relevant not only at a diagnostic level, but also from a prognostic perspective. Further research on the prognostic role of LI in this population is thus worthwhile, also within the longitudinal dimension [47]—and, thus, by advisably addressing technology-aided language assessment procedures that, as fully overcoming motor limitations, are feasible across all disease stages [48–50].

Finally, as far as the covariates entered within the NBRs, this study disclosed a number of incidental findings that are worth a tentative explanation. First, younger age unexpectedly proved to be inversely related to both BADL and IADL scores. Such a result is highly controversial, given that older age has been typically linked to a greater degree of functional dependence [51]. However, both the BADL and the IADL have been reported to be possibly biased by age [52–55]—with some items being more likely to be endorsed by younger individuals and others by older ones. Hence, it is likely that the present findings regarding age might be measurement-specific, rather than reflecting an actual association between this demographic and FI in ALS. A similar explanation might be applicable to the finding of female sex being associated with lower BADL scores, since sex biases in the ADL scales herewith employed have been described as well—i.e. some items being more likely to be endorsed by males and others by females [52, 53, 55]. As to the association between higher STAI-Y2 scores and higher BADL scores, a mediating role of apathetic features might be advanced. In fact, an inverse association has been recently reported in this population between anxiety—albeit a state-level—and apathy—albeit as far as emotional disintegration is concerned [23]. Since apathy has been herewith found detrimentally affect FI, it might be postulated that patients with a greater degree of apathetic features, and thus of functional dependence, were at the same time features by lower arousal—and thus anxiety—levels. Nevertheless, such an explanation is merely speculative and not empirically supported on the basis of the current results. Whilst it would be far beyond the aim of this study to explore the interplay between apathy, anxiety and FI in ALS, this matter might explored in future studies.

This study is of course not free of limitations. First, FI was herewith operationalized via aspecific scales that are known to tap on cognitive-driven ADL to a limited extent [56]. Hence, future studies on the association between FTSDs and FI in this population should address ADL scales which heavily load on cognitive functioning—such as the Amsterdam IADL Questionnaire© [57], which has shown excellent clinimetrics and feasibility in neurodegenerative disorders [57–61]. Second, within the present study, cognition has been evaluated by means of a screening test—i.e. the ECAS: although this test has been thoroughly shown to be clinimetrically sound and feasible within the Italian scenario [12, 47, 63, 64], it is undoubtedly advisable that future studies further investigate the interplay between FTSDs and FI in this population by employing domain-/function-specific, second-level tests. Relatedly, it has to be mentioned that the ECAS-L has been criticized as not being able to fully cover the spectrum of ALS patients’ language phenotypes [45, 62, 63]: thus, future investigations should aim at replicating—or disconfirming—the present findings by employing a detailed and extensive set of second-level language measures. Finally, behavioural features were herewith assessed via both a caregiver-report—i.e. the BBI—and a self-report scale—i.e. the DAS. Such a discrepancy related to the source of information on patients’ behavioural status might have altered, at least to some extent, the current findings. It is thus advisable that future investigations on the topic address a consistent source of information.

In conclusion, among FTSDs, language changes might affect instrumental ADL in non-demented ALS patients, whilst apathetic features—and, more specifically, a diminished cognitive-behavioural initiation—might impact on basic ADL in this population.

## Data Availability

Datasets associated with the present study have been stored on an online repository (10.5281/zenodo.8384038) and are available upon reasonable request of interested researchers.
